# TPH1 inhibits bladder tumorigenesis by targeting HIF-1α pathway in bladder cancer

**DOI:** 10.2478/abm-2024-0023

**Published:** 2024-09-20

**Authors:** Jianwei Ren, Zhiting Mo, Xia Deng, Minghui Ren, Hailong Ren, Jie Jin, Huihui Zhang

**Affiliations:** Department of Basic Medicine, Tibet University Medical College, Lhasa, Tibet 850000, China; Department of Clinical Laboratory, Lhasa People’s Hospital, Lhasa, Tibet 850000, China; The Brain Cognition and Brain Disease Institute, Shenzhen Institute of Advanced Technology, Chinese Academy of Sciences; Shenzhen-Hong Kong Institute of Brain Science-Shenzhen Fundamental Research Institutions, Shenzhen, Guangdong 518055, China; School of Basic Medical Sciences, Wuhan University, Wuhan, Hubei 430072, China; Department of Laboratory Medicine, Hunan Normal University School of Medicine, Changsha, Hunan 410013, China

**Keywords:** bladder cancer, HIF-1α pathway, T24, TPH1, tumorigenesis

## Abstract

**Background:**

BCa is the most common cancer of the urinary system. TPH1 has been reported to be associated with distinct tumorigenesis. However, the role of TPH1 in BCa remains to be clarified.

**Objectives:**

Our aim is to demonstrate the molecular mechanism of TPH1 in BCa carcinogenesis and development.

**Methods:**

In research, we explored the effect of TPH1 on T24 cells. Colony formation, soft agar, and cell proliferation assays were used to determine the survival and proliferative capacity of cells. Moreover, TPH1^−/−^ cell lines were analyzed using *CRISP-CAS9*, and the recovery experiment was conducted. Realtime fluorescence quantitative PCR (qPCR) and Western blot were used to detect HIF-1α mRNA levels and TPH1 protein.

**Results:**

The TPH1 expression is lower in tumor tissues than in normal tissues. Colony formation, soft agar, and cell proliferation assays revealed that the overexpression of TPH1 declined cells survival. Moreover, the deficiency of TPH1 increased the number of clones. These results suggested that survival rate of TPH1 overexpression was repressed in cells. In addition, we found that HIF-1α activity was significantly downregulated with an increase in TPH1. The transcriptional activity of survivin was increased with TPH1^−/−^ cells. Then, the proliferative ability of TPH1^−/−^ cells was almost similar to the wild type levels with the treatment of LW6, TPH1 might play a major role to repress HIF-1α activity.

**Conclusions:**

Taken together, these results suggested that increasing TPH1 activity could inhibit survival and proliferation of cells via HIF-1α pathway. TPH1 may be a potential target for human BCa therapy.

Bladder cancer (BCa) is one of the most common malignancies of the urinary system, which directly threatens the health and lives of patients. Monitoring and evaluating the cancer incidence rate, mortality rate, and disability adjusted life year involving 195 countries from Global Burden of Disease (GBD) indicated that the incidence rate of BCa ranks among the top 10 worldwide with 474,000 new cases and 197,000 deaths globally in 2017. BCa incidence of men (1/74) was more than four times than women (1/301) over a lifetime [1]. Of all the types of BCa, the cases of transitional epithelium cell carcinoma (BTCC) accounted for about 90%. BTCC has two subtypes: muscle invasive bladder cancer (MIBC) (about 20%) and non-muscle invasive bladder cancer (NMIBC) (about 70%–80%). The former is associated with higher mortality, and the latter with a low progression rate but a high incidence of recurrence. Thus, although nonfatal, NMIBC patients require long-term monitoring and regular surveillance, which makes it more difficult and expensive to cure them [2, 3].

Hypoxia-inducible factor-1 alpha (HIF-1α) is an important transcription factor with extensive distribution and functions in mammalian tissue cells. HIF-1 consists of two subunits: oxygen-sensitive HIF-1α and oxygen-insensitive HIF-1β. HIF-1α plays major roles in transcription and regulation of tissues under hypoxia condition [2, 4]. Previous research had shown that overexpression of HIF-1α has been observed in multiple types of tumors, such as liver cancer, breast cancer, brain cancer, BCa, and other tumor tissues, which can be regulated by tumor-related signaling. It was reported that the overexpression of HIF-1α in BCa can activate >1,000 genes, such as survivin, under hypoxia condition [5].

Tryptophan hydroxylase (TPH) is the rate-limiting enzyme involved in serotonin synthesis. It has two subtypes, namely, TPH1 and TPH2, encoded by two genes located on different chromosomes. With 71% sequence similarity, the same biological activity, and similar substrate specificity, they have different phosphorylation sites and finally cause different physiological effects [6,7,8]. TPH1, the source of most peripheral serotonin and the key enzyme of peripheral serotonin synthesis, is mainly found in the epithelial tissues of the digestive tract and gastrointestinal tract, but it is also found in adrenal glands, kidneys, and the pineal gland tissues. By contrast, TPH2 is mainly found in neurons of the central nervous system [9, 10].

TPH1 and TPH2 are involved not only in development, cardiovascular function, bone homeostasis, gastrointestinal motility, hemostasis, and liver regeneration but also in tumorigenesis. Gautam et al. [11] found that the level of TPH1 expressed by triple-negative breast cancer (TNBC) cell lines was higher than that of hormone-responsive breast cancer cell lines, revealing that the effect of 5-hydroxytryptamine (5-HT) on the progression of breast cancer is due to TPH1 that promotes the production of 5-HT. Li et al. [12] found that 5-HT levels, as well as the expression of TPH1, were significantly upregulated in colorectal tumor tissues when compared with normal colorectal tissues or epithelial cell lines. Silencing TPH1 expression slowed down tumor growth in an established mouse model, whereas treatment with TPH1 inhibitor alleviated tumor progression in an azoxymethane/dextran sodium sulphate (AOM/DSS)-induced colorectal cancer (CRC) mouse model.

Kim et al. [13] found that Carnitine O-palmitoyltransferase 1 (CPT1B), Palmitoyl thioesterase CPT1C (CPT1C), Mitochondrial carnitine/acylcarnitine carrier protein (SLC25A20), Carnitine O-acetyltransferase (CRAT), TPH1, and Type I iodothyronine deiodinase (IOD1) were significantly downregulated in tumor tissues using reverse transcription PCR (RT-PCR) compared with normal bladder tissues of patients with NMIBC and TPH1 were downregulated in those with MIBC. However, the mechanism is still not clear. We had speculated that the decrease of TPH1 transcriptional activity might be related to the tumorigenesis of bladder tissues; moreover, TPH1 was more likely to have an inhibitory effect on BCa development.

## Materials and methods

### Cell culture, plasmid transfections, and treatment with HIF-1α inhibitor

Human BCa T24 cell line used in this study was purchased from American Type Culture Collection (ATCC, HTB-4^TM^). Cells were cultured in RPMI-1640 medium (HyClone) supplemented with 10% fetal bovine serum (HyClone). For transfection, cells were transfected with TPH1 recombinant plasmids labeled FLAG or Clustered Regularly Interspaced Short Palindromic Repeats-CRISPR-associated protein 9 (CRISPER–Cas9) system targeting sequence using Lipofectamine^TM^ 2000 transfection reagent (Thermo Fisher Scientific) according to the manufacturer’s instructions. LW6(HIF-1α inhibitor), a novel HIF-1α inhibitor, was purchased from MCE (MedChemExpress). LW6 was added to RPMI-1640 medium at 5 μM for 12 h after T24 cells were cultured at 37°C for 24 h.

### Establishment of knockout cell line

The CRISPR-CAS9 technique was used to construct TPH1-knockout BCa cell line in T24 cells following the Zhang Lab protocol [14]. Small guide RNA (sgRNA) was designed using the CRISPR design tool. The sgRNA sequence was 5′-TGAAAATCTTTCAGGTAAGC-3′. sgRNA guide sequences can be cloned into an expression plasmid containing sgRNA scaffold backbone. The other expression plasmid bearing Cas9 was ready to use. Both sgRNA plasmid and Cas9 plasmid were transfected into cells. Positive cells were selected by puromycin (1 μg/mL). Western blot analysis was used for cell line identification.

### Protein extraction and Western blot analysis

Western blot analysis was prepared as described [15]. In brief, T24 cells were cultured in 6-well plates and subsequently transfected with indicated plasmid for 36 h as the confluency reached 60%. T24 cells were washed with phosphate buffer saline (PBS)for three times and lysed with SDS sample buffer (62.5 mM Tris–HCl, pH 6.8, 2% SDS, and 10% glycerol) at 95°C for 10 min. Protein concentration was determined by using a BCA protein assay kit (Thermo Fisher Scientific). Proteins were separated using 15% SDS-PAGE gels and transferred to Polyvinylidene fluoride (PVDF) membranes (IPVH00010; Millipore). The membranes were blocked with 5% non-fat milk and incubated with primary antibodies overnight at 4°C. After washing with tris buffered saline with tween 20 (TBST), the membrane was incubated with horseradish peroxidase (HRP)-conjugated secondary antibodies at room temperature for 1 h. Protein expressions were visualized using ChemiDocTM MP Imaging System (Bio-Rad). The GAPDH was used as a loading control.

### Colony formation, soft agar, and CCK-8 assays

Colony formation, soft agar, and Cell Counting Kit-8 (CCK-8) assays were used to examine the cell viability and proliferation. Briefly, after 48 h transfection, cells were seeded in 6-well plates (400 cells/well). After 14 days, colonies were stained with 0.05% crystal violet at room temperature for 30 min. Images were captured by a scanner (MRS-2400U2; Microtek). The number of colonies was counted and analyzed statistically. For soft agar assays, 2 mL of 0.7% lower agar was plated onto 6-well plates. A volume of 1 mL T24 cells (5 × 10^4^) was mixed with 1 mL of 0.7% agar and added to curdled lower agar; 2 mL medium was added to upper agar; and the 6-well plates were incubated at 37°C in 5% CO_2_ for about 3 weeks. Ultimately, the number of clones was photographed and counted. CCK-8, an alternative one for Thiazolyl Blue (MTT) method, was used to determine cell viability. Notably, 1 × 10^4^ T24 cells/well were seeded into 96-well plates. Then, 10 μL of CCK-8 reagent solution was added to each well and cultured for 1–3 h. Then, the absorbance value OD (optical density) was measured in a microplate reader at the wavelength of 450 nm. The cell viability curve with the time as abscissa (x-axis) and OD value as ordinate (y-axis) was determined. The procedures were repeated three times independently to obtain a mean value OD_450_.

### RNA extraction and quantitative real-time PCR

Total RNA was extracted using RNAiso Plus (9108, Takara Biomedical Technology (Beijing) Co., Ltd) according to the manufacturer’s instructions. The reverse transcription reaction was carried out according to the manufacturer’s instructions (Roche). Specific primers (survivin: forward 5′-TTCTCAAGGACCACCGCATC-3′ and reverse 5′-GCCAAGTCTGGCTCGTTCTC-3′) were used for real-time PCR.

### Luciferase reporter test

Cells were seeded into 24-well plates and transfected with the indicated plasmids. The reporter plasmid (200 ng/well), pRL-CMV (2 ng/well), and gene expression plasmid were used in each transfection. After 36 h, reporter assays were performed with a dual-specific luciferase assay kit (Promega).

### Statistical analysis

The experimental data were displayed as mean ± SEM; each experiment was repeated three times; and *P* < 0.05 was considered to be significant.

## Results

### Association of low expression of TPH1 in tumor tissues with poor prognosis

**Figure 1.**
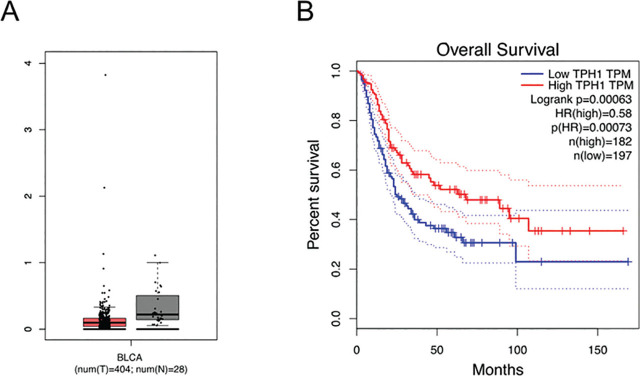
TPH1 was associated with carcinogenesis of bladder tissues. **(A)** Analysis of TPH1 average expression level in bladder tissues. BCa tissues of 404 cases and normal bladder tissues of 28 cases were collected. **(B)** The effect of different expression levels of TPH1 on the survival rate. BCa, bladder cancer; TPH1, tryptophan hydroxylase 1.

To explore the effect of TPH1 in BCa tumorigenesis, we initially analyzed the expression of TPH1 in BCa and normal tissues using the interactive web server GEPIA (http://gepia.cancer-pku.cn/). The average expression level of TPH1 was lower in BCa tissues (n = 404 cases) compared with normal tissues (n = 28 cases) (**Figure 1A**). Furthermore, the survival percentage of patients varied with time as the TPH1 expression level changed; patients with higher expression of TPH1 are more likely to survive than those with lower expression (**Figure 1B**). From the results above, low expression of TPH1 is associated with poor prognosis and reduces the mortality rate.

### TPH1 inhibits T24 cells viability and proliferation

**Figure 2.**
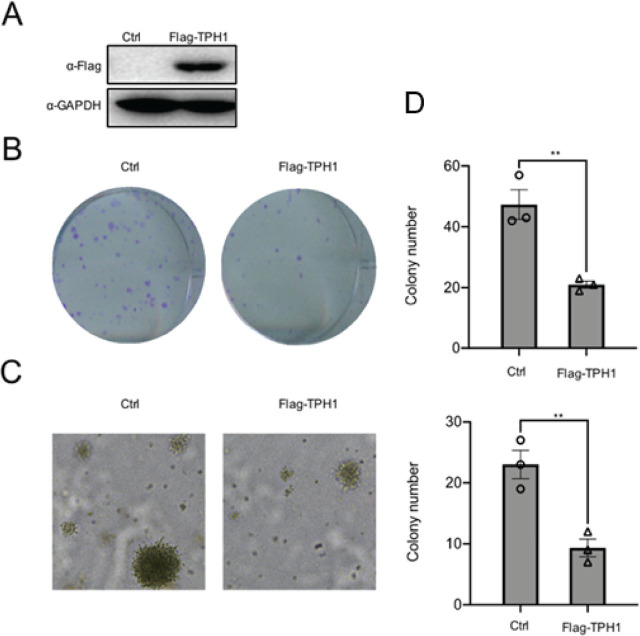
TPH1 inhibited the growth and proliferation of T24 cells. **(A)** The overexpression of TPH1 protein was determined by using Western blot analysis. **(B)** T24 cells were transfected with Flag-TPH1 plasmids, and untreated T24 cells were used as the control. Colony formation assays were performed to detect cell viability. A total of 4 × 10^2^ cells were seeded in 6-well plates. After 14 days, cells were fixed with 4% polyformaldehyde and stained with 0.05% crystal violet. The number of cell clones was counted. (**C**) Soft agar assays were used to evaluate the proliferation of cells with overexpression of TPH1. (**D**) CCK-8 assays were performed to determine cell viability, and the procedures were repeated three times to obtain a mean value. Values are expressed as mean ± SEM. ^**^P < 0.01. CCK-8, Cell Counting Kit-8; TPH1, tryptophan hydroxylase 1.

To investigate the effect of TPH1 on cell proliferation, a recombinant plasmid Flag-TPH1 was constructed. The plasmids were transfected into T24 cells, and then the protein was extracted after 36 h. Subsequently, protein level was detected using Western blot analysis (**Figure 2A**). To analyze the function of TPH1, colony formation assay was performed, and the number of cell colons was counted. The results revealed that overexpression of TPH1 repressed human BCa T24 cells viability (**Figure 2B**). In addition, soft agar colony formation assays also proved the same conclusion (**Figure 2C**), which further indicated that the overexpression of TPH1 inhibited the growth and proliferation of human BCa T24 cells. Compared with the control, survival rate of cells was decreased with time-dependent variation due to the transfection of TPH1 (**Figure 2D**), which showed that the exogenous overexpression of TPH1 suppressed T24 cell viability.

### TPH1-knockout T24 promotes cell survival and proliferation

**Figure 3.**
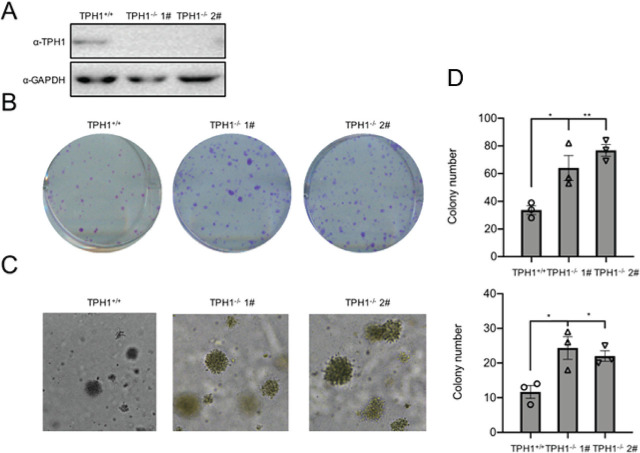
The deficiency of TPH1 promoted cell survival and proliferation. (**A**) Western blot analysis was used to analyze TPH1 protein expression in WT and TPH1-knockout T24 cells. (**B**) Colony formation assays showed the viability of TPH1-knockout T24 BCa cells. Cells were digested and seeded into 6 cm plates. After 2 weeks, colonies were stained with crystal violet, imaged, and counted. (**C**) Soft agar assays were used to evaluate the proliferation of WT and TPH1-knockout T24 BCa cells. The colonies were imaged and counted. (**D**) CCK-8 assays were performed to determine cell viability, and the procedures were repeated three times to obtain a mean value. Values are expressed as mean ± SEM. ^*^P < 0.05, ^**^P < 0.01, ^***^P < 0.001. BCa, bladder cancer; CCK-8, Cell Counting Kit-8; TPH1, tryptophan hydroxylase 1; WT, wild type.

To further confirm the effect of TPH1 gene knockout on cell proliferation, TPH1-knockout T24 cells were established using the CRISPER–Cas9 system. Subsequently, protein levels were confirmed using Western blot analysis, and two colonies (1# and 2#) were selected (**Figure 3A**). Colony formation assays revealed that the survival rate and proliferative ability of TPH1-knockout T24 cells increased obviously compared with the wild-type (WT) cells (**Figure 3B**). More and larger cell clones of TPH1-knockout cells were photographed and counted with soft agar assays (**Figure 3C**). CCK-8 assays showed that the deficiency of TPH1 potently increased cell proliferation (**Figure 3D**). These results confirmed that the deletion of TPH1 gene promoted the survival and proliferation of human BCa T24 cells, and it may also promote the formation of BCa solid tumor.

### Effects of TPH1 on HIF-1α signal pathway

**Figure 4.**
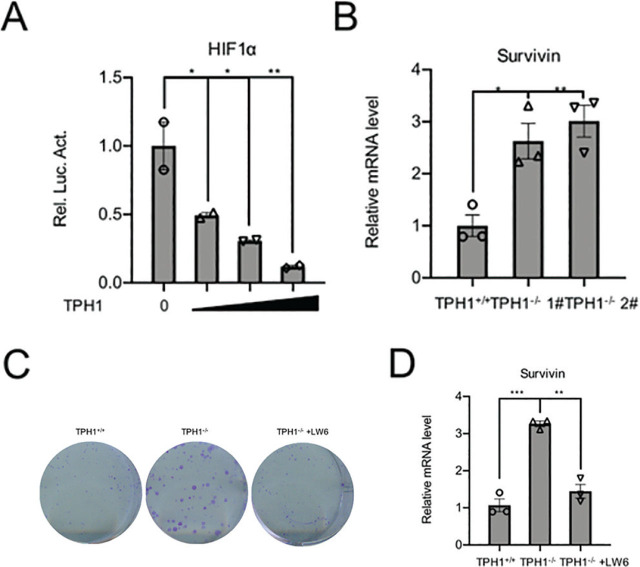
TPH1 affected the survival and proliferation of T24 cells by regulating HIF-1α signal pathway. (**A**) Luciferase assays were used to detect the activity of HIF-1α pathway with increasing amounts of the TPH1 expression plasmids (0 ng, 100 ng, 200 ng, and 400 ng). (**B**) RT-PCR was used to analyze the survivin mRNA levels in WT and TPH1-knockout cells. (**C**) Colony formation showed the viability of TPH1-knockout T24 BCa cells treated with or without LW6. (**D**) RT-PCR was used to analyze the survivin mRNA levels in TPH1-knockout cells treated with or without LW6. Values are expressed as mean ± SEM. ^*^P < 0.05, ^**^P < 0.01, ^***^P < 0.001. BCa, bladder cancer; HIF-1α, hypoxia-inducible factor-1 alpha; TPH1, tryptophan hydroxylase 1; WT, wild type.

To explore the molecular mechanism of TPH1 inhibiting the growth and proliferation of BCa T24 cells, we performed luciferase pathway screening and found that TPH1 overexpression decreased HIF-1α activity. Furthermore, luciferase assays showed that transcriptional activity of HIF-1α decreased gradually in a concentration-dependent manner of TPH1, and there was a significant difference between different concentration gradients (*P* < 0.05) (**Figure 4A**). Survivin, a downstream signaling molecule of HIF-1α, is a strong inhibitor of apoptosis, which can be regulated positively by HIF-1α and expressed in common malignant tumors, such as breast cancer, BCa, renal cancer, colorectal cancer, neuroblastoma, and ovarian cancer, but not in normal tissues. The mRNA level of survivin was increased in TPH1-knockout cells (**Figure 4B**). In other words, TPH1 influenced BCa cell behavior by regulating HIF-1α-associated pathway activity. To confirm whether TPH1 affected cell survival through HIF-1α, we treated TPH1-knockout cells using HIF-1 inhibitor LW6 at 5 μM. The results showed that the number of cell clones returned to the same level as those of WT cells after the inhibition of HIF-1α signaling (**Figure 4C**). RT-PCR assays revealed that the mRNA level of survivin decreased in TPH1^−/−^ cells after LW6 was added (**Figure 4D**). The above results indicated that TPH1 could inhibit the proliferation of human BCa T24 cells via HIF-1α pathway.

## Discussion

TPH, as the rate-limiting enzyme in serotonin synthesis, could convert tryptophan to 5-hydroxytryptophan (5-HTP), which is subsequently decarboxylated to form serotonin. TPH exists in two isoforms: TPH1 and TPH2. TPH1 is usually expressed only in the thymus, spleen, and enterochromaffin cells of the gut, while TPH2 is generally confined to the brain stem [16, 17]. TPH1 gene encodes a member of the aromatic amino acid hydroxylase family. The encoded protein catalyzes the first and rate-limiting step in the biosynthesis of serotonin, an important hormone and neurotransmitter. If TPH1 mutation occurs, it is closely related to human neuropsychiatric diseases such as personality disorder, schizophrenia, emotion regulation, depression, and even intelligence quotient. Recently, some reports have shown that TPH1 also plays a role in cancer progression [12, 17,18,19,20,21]. Li et al. [12] found that 5-HT levels, as well as the expression of TPH1, were significantly upregulated in colorectal tumor tissues collected from patients with colorectal cancer and colorectal cancer cell lines compared with normal colorectal tissues or epithelial cell lines. 5-HT enhanced NLRP3 (NACHT, LRR and PYD domains-containing protein 3) inflammasome activation in immortalized bone marrow-derived macrophages via its ion channel receptor, HTR3A (5-hydroxytryptamine receptor 3A). Liu et al. [19] demonstrated that Signal transducers and activators of transcription 5 (STAT5) in CD8^+^ T cells was activated with a high level of Interleukin-2 (IL-2), which in turn induced strong expression of TPH1, thus catalyzing the conversion to tryptophan to 5-hydroxytryptophan (5-HTP). 5-HTP subsequently activated AhR nuclear translocation, leading to a downregulation of cytokine and effector molecule and thereby causing T cells dysfunction in the tumor microenvironment. Nakagawa et al. [20] demonstrated that silencing of androgen–androgen receptor signaling may cause initiation and progression of seminomas through an increase in TPH1 gene expression level via the activation of androgen–androgen receptor signaling in seminoma cells.

BCa is one of the most common tumors of the urinary system. Similar to other tumors, BCa is a complex process involving the interaction between multiple factors and multiple procedures. It is important to elucidate the mechanism and its influential factors. Recent evidence suggests that the hypoxic environment in tumors is a common phenomenon that could lead to tumorigenesis by activating the major transcription factor. As a key transcription factor of the adaptive response to hypoxia, HIF-1α overexpression is related to the adverse prognosis and mortality [22,23,24,25].

There are only few studies on the mechanism of regulating HIF-1α in BCa. Recent studies have illustrated that the expression of microRNAs, which could bind to the 3′ untranslated region of the target genes by complementary pairing, is regulated by hypoxia. For example, the oncogenic role of Long Non-coding RNA Cancer Susceptibility Candidate 9 could promote the progression of nasopharyngeal carcinogenesis by stabilizing HIF-1α [26]. Qiu et al. [27] demonstrated the role and clinical relevance of miRNA-138 and HIF-1α in melanoma cell growth and metastasis, and pointed out that microRNA-138 could suppress melanoma growth and metastasis via negatively regulating HIF-1α. Byun et al. [28] showed that MiR-200c could negatively affect HIF-1α and migration of lung cancer cells. Recent study had shown that Histone-lysine N-methyltransferase SETD1A/Sentrin-specific protease 1 (SETD1A/SENP1) could regulate glycolysis and modulate the progression of gastric cancer and prostatic carcinoma cells by interacting with HIF-1α, which implicated that SETD1A/SENP1 may be a novel molecular target for the gastric cancer and prostatic carcinoma treatment [29, 30].

TPH1 has been reported to be involved in BCa, which were significantly downregulated in BCa tissues compared with normal bladder tissues of patients with NMIBC [13]. But the effect of TPH1 on the biological behaviors of BCa is still unclear. In our research, we constructed FLAG-TPH1 recombinant plasmids and transfected into T24 cells. Colony formation and soft agar assays showed that cell colonies declined in number and CCK-8 assays proved that the T24 cell viability decreased, indicating that the overexpression of TPH1 would inhibit cell proliferation. Sequence specifically targeting TPH1 was used to knock out TPH1 gene. In addition, we constructed TPH1-knockout T24 cells using CRISPR–Cas9 system. Colony formation and soft agar assays suggested increases in the number of cell colonies and cell viability which suggest that the deletion of TPH1 could promote cell proliferation. To study the molecular mechanism of TPH1 in tumorigenesis, luciferase assays showed that the transcription activity of HIF-1α decreases with increasing amount of TPH1. In addition, real-time PCR assays suggested the transcription activity of survivin was upregulated in TPH1-knockout T24 cells. Colony formation assays proved that the proliferation ability of TPH1-knockout cells decreased than that of the WT level after treating with HIF-1α inhibitor LW6, which was in agreement with its facilitated proliferation function. The PCR results revealed that the mRNA levels of survivin in TPH1-knockout cell line were restored to that of the WT level after adding HIF-1α inhibitor LW6. We speculated that TPH1 is located upstream of HIF1 and can inhibit the transcriptional activity of HIF1. These results prove that TPH1 can regulate cell viability and growth by regulating HIF-1α signaling pathway.

In conclusion, we explored the interrelation between TPH1 and HIF-1α signal pathway in T24 BCa cell line by using various molecular experiments. Through clone formation assays, growth curve, Western blot analysis, gene knockout, and other experiments, it was confirmed that TPH1 could inhibit BCa development via HIF-1α pathway. TPH1 gene is expected to become a novel target for the treatment of BCa.
